# Supplementary material for: Induced Effect of Environmental Regulation on Green Innovation: Evidence from the Increasing-Block Pricing Scheme
Source: Int J Environ Res Public Health. 2021 Mar 5;18(5):2620. doi: 10.3390/ijerph18052620 (PMC7967318; doi:10.3390/ijerph18052620)
Supplement: Supplementary file 1 [file ijerph-18-02620-s001.pdf]

Table S1 Covariates

Covariate	Definition	Measurement	Sources
Technological patents	Logarithm of technological patents	count	the *State Intellectual Property Office*
Energy-efficient patents	Logarithm of energy-efficient patents	count	the *State Intellectual Property Office*
Level of economy	Logarithm of GDP	hundred million	the *China Statistical Yearbook*
Economic structure	Ratio of secondary industry to service sector	percent	the *China Statistical Yearbook*
Economic environment	Proportion of scaled firms at a loss in all firms	percent	the *China Statistical Yearbook*
International trade	Ratio of FDI to total GDP	percent	the *China Statistical Yearbook*
Human resources	Average education years of citizens over the age of 6	year	the *Easy Professional Superior* database
Investment in R&D	Logarithm of the internal expenditures on R&D	hundred million	the *Easy Professional Superior* database

Table S2 Detail of IPC Green Inventory topics and sub-topics covered related to technological innovation, among which energy-efficient related topics are shown in bold.

Topic	Sub-topics			
Alternative Energy Production	Bio-fuels	Solid fuels	Torrefaction of biomass	
		Liquid fuels	Vegetable oils	
			Biodiesel	
			Bioethanol	
		Biogas		
		From genetically engineered organisms		
	Integrated gasification combined cycle (IGCC)			
	Fuel cells	Electrodes	Inert electrodes with catalytic activity	
		Non-active parts		
		Within hybrid cells		
	Pyrolysis or gasification of biomass			
	Harnessing energy from manmade waste	Agricultural Waste	Fuel from animal waste and crop residues	
			Incinerators for field, garden or wood waste	
		Gasification		
		Chemical waste		
		Industrial	Using top gas in blast furnaces to power pig-iron production	
			Pulp liquors	
			Anaerobic digestion of industrial waste	
		Hospital waste		

		Landfill gas	Separation of components	
		Municipal waste		
	Hydro energy	Water-power plants	Tide or wave power plants	
		Machines or engines for liquids	Using wave or tide energy	
		Regulating, controlling or safety means of machines or engines		
		Propulsion of marine vessels using energy derived from water movement		
	Ocean thermal energy conversion (OTEC)			
	Wind energy	Structural association of electric generator with mechanical driving motor		
		Structural aspects of wind turbines		
		Propulsion of vehicles using wind power	Electric propulsion of vehicles using wind power	
		Propulsion of marine vessels by wind-powered motors		
		**Photovoltaics (PV)**	Devices adapted for the conversion of radiation energy into electrical energy	Using organic materials, the active part
			Assemblies of a plurality of solar cells	
			Silicon; single-crystal growth	
			Regulating to the maximum	

			power available from solar cells	
			**Electric lighting devices with, or rechargeable with, solar cell**	
			**Charging batteries**	
			Dye-sensitised solar cells(DSSC)	
		Use of solar heat	For domestic hot water systems	
			For space heating	
	Solar energy		For swimming pools	
			Solar updraft towers	
			For treatment of water, waste water or sludge	
			Gas turbine of water, waste water or sludge	
		Hybrid solar thermal-PV systems		
		Propulsion of vehicles using solar power	Electric propulsion of vehicles using solar power	
		Producing mechanical power from solar energy		
		**Roof covering aspects of energy collecting devices**		
		Steam generation using solar heat		
		**Refrigeration or heat**		

		**pump systems using solar energy**		
		Use of solar energy for dying materials or objects		
		Solar ponds		
	Geothermal energy	Use of geothermal heat		
		Production of mechanical power from geothermal energy		
	Other production or use of heat, not Derived from combustion, e.g. natural heat	Heat pumps in central heating systems using heat accumulated in storage masses		
		Heat pumps in other domestic-or spaceheating systems		
		Heat pumps in domestic hot-water supply systems		
		Air or water heaters using heat pumps		
		Heat pumps		
	Using waste heat	To produce mechanical energy		
		Of combustion engines		
		Of steam engine plants		
		Of gas-turbine plants		
		As source of energy for refrigeration plants		
		For treatment of water, waste water or		
		Recovery of waste heat in paper production		
		For steam generation by exploitation of the		
		heat content of hot heat carriers		
		Recuperation of heat energy from waste		
		incineration		
		Energy recovery in air		

		conditioning		
		Arrangements for using waste heat from		
		Regenerative heat-exchange apparatus		
		Of gasification plants		
	Devices for producing mechanical power from muscle energy			
Transportation	Vehicles in general (e.g. hybrid vehicles, electric propulsion)	Hybrid vehicles, e.g. Hybrid Electric Vehicles (HEVs)	Control systems	
			Gearings therefor	
		Brushless motors		
		Electromagnetic clutches		
		Regenerative braking systems		
		Electric propulsion with power supply from		
		force of nature, e.g. sun, wind		
		Electric propulsion with power supply	With power supply from fuel cells, e.g. for hydrogen vehicles	
		Combustion engines operating on gaseous		
		fuels, e.g. hydrogen		
		Power supply from force of nature,		
	Vehicles other than rail vehicles Drag reduction	Human-powered vehicle		
		Drag reduction		
	Rail vehicles	Drag reduction		
	Marine vessel propulsion	Propulsive devices directly acted on by wind		
		Propulsion by wind-powered motors		

		Propulsion using energy derived from water movement		
		Propulsion by muscle power		
		Propulsion derived from nuclear energy		
	Cosmonautic vehicles using solar energy			
Energy conservation	**Storage of electrical energy**			
	**Power supply circuitry**	With power saving modes		
	**Measurement of electricity consumption**			
	**Storage of thermal energy**			
	**Low energy lighting**	Electroluminescent light sources (e.g. LEDs, OLEDs, PLEDs)		
	**Thermal building insulation, in genera**	Insulating building elements	For door or window openings	
			For walls	
			For floors	
			For roofs	
			For ceilings	
	**Recovering mechanical energy**	Chargeable mechanical accumulators in		
Nuclear power generation	Nuclear engineering	Fusion reactors		
		Nuclear (fission) reactors		
		Nuclear power plant		
	Gas turbine power plants using heat source of nuclear origin			

**Table S3** Balance of key determinants between treated group and control group				
Covariate	Treated group	Control group	Unconditional diff.	Conditional diff.
Residential electricity consumption in 1999	368.553 [161.287]	253.318 [112.137]	0.520 (0.231)	
Level of economy in 1999	8.328 [0.252]	7.622 [0.912]	0.575*** (0.113)	0.007 (0.027)
Economic structure in 1999	0.829 [0.173]	0.9042 [0.289]	0.301*** (0.050)	0.151 (0.494)
Economic environment in 1999	0.275 [0.093]	0.026 [0.100]	-1.223*** (0.551)	-0.094 (0.093)
International trade in 1999	0.034 [0.035]	0.026 [.025]	-3.724*** (0.493)	-2.134 (-2.556)
Human resources in 1999	7.604 [0.775]	8.068 [1.097]	0.031 (0.890)	0.001 (0.004)
Investment in R&D in 1999	7.413 [0.680]	6.895 [1.309]	0.384*** (0.105)	0.06 (0.080)

Note: This table reports the summary statistics of our treatment and control samples. First line of results the comparison of selection criteria between the treatment and control groups. Following results are comparisons between the treatment and control groups on various economic and techonological variables in the initial year, both before and after controlling for the selection criteria. Columns 1 and 2 show means and standard deviations in square brackets. Column 3 reports the unconditional difference between the treatment and control group. Column 4 reports the conditional difference of these characteristics of a regression on the treatment dummy controlling for the selection criteria. The standard errors are reported in parentheses.

Table A4 Robustness test

	(1)	(2)	(3)	(4)
Variables	All technological patents	Energy-efficient patents	Ratio	Ratio
IBP	4.312**	0.383***	0.268**	-1.164*
	(2.18)	(3.19)	(2.44)	(-1.78)
Level of economy	3.503	0.306	0.543**	0.724
	(0.95)	(1.35)	(2.50)	(1.22)
Economic structure	5.546***	0.381	0.029	-0.175
	(3.48)	(3.05)	(0.28)	(-0.50)
Economic environment	2.822	-0.362***	-0.123***	-4.098*
	(0.40)	(-0.78)	(-3.51)	(-1.75)
International trade	-9.046*	-4.720	0.05	-1.835
	(-1.74)	(-1.61)	(0.29)	(-0.34)
Human resources	0.478	0.026	-0.006	0.225**
	(1.15)	(0.98)	(-0.33)	(2.27)
Investment in R&D	3.992*	0.251	-0.014	-0.887**
	(-2.76)	(1.61)	(-0.95)	(-2.39)
Constant	6.320***	4.651***	-0.249	7.576**
	(-2.76)	(-3.45)	(-3.29) ***	(2.19)
Observation	150	150	150	232

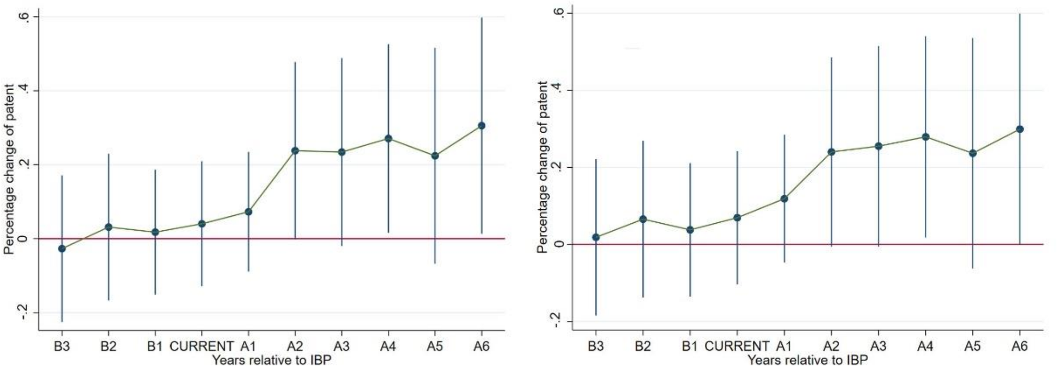

(a) Energy-efficient patents

(b) The ratio of energy-efficient patent to all patents

Fig. S1 The pre-existing trend test of the IBP on patents.
